# The Practice of Medicine

**Published:** 1897-09

**Authors:** 


**The Practice of Medicine.** By HORATIO C. WOOD, M.D., and REGINALD H. FITZ, M.D. Pp. x., 1088. Philadelphia: J. B. Lippincott Company. 1897.

The aim of this work is to view the practice of medicine simultaneously from the pathologic and therapeutic points of view. All the therapeutic portions of the book have been written by Dr. Wood; the more especially pathological parts are from the pen of Dr. Fitz. These two professors, the one of Therapeutics at Pennsylvania, the other formerly of Pathology at Harvard, have produced a joint volume, which may be well epitomised by the motto on the title page, "Droit et Avant."

Nothing is more difficult than to give an adequate account of a text-book on medicine: one can only mention such points as fall under one's notice in a general survey, and these points are more likely to be errors of omission than inaccuracies of a positive kind. There is one such in the description of scarlet fever, where we read that "in order to allay the burning and itching of the skin, cosmoline, cacao butter, cold cream, lard freed from salt by washing, olive oil, or other bland fat should be freely applied to the surface of the body morning and evening, after the first or second day of the eruption." Surely this is an altogether inadequate description of the practice of inunction in scarlet fever, which has a more important purpose to serve than the mere sedative action on the skin: the authors add no antiseptics and make no attempt to prevent the dissemination of active germs beyond the mere mechanical operation of preventing dried scales from flying about. We have for years been under the impression that antiseptic inunction was our principal safeguard in limitation of the disease, and that with care we need never have a second case, as no contagious disease seems to be more under control than this one, inasmuch as there is usually time to isolate and disinfect before the time when dissemination is likely to occur. The authors give more attention to the disinfection of the sick room, but they disregard the patient himself, whereas we hold that if the patient is well disinfected by inunction and bathing, there will be less need for some of the details of disinfection of the patient's surroundings.

Under typhoid fever we fail to find any mention of Widal's bacteriological test, which is becoming so much more useful than the test of Elsner; the diazo-reaction of Ehrlich is described as "of limited value in the recognition of typhoid fever." We have become accustomed to think that the diagnosis of the disease is not complete without the evidence of these two tests, and that if they give a negative result the case is likely to be one of meningitis or tuberculosis. We cannot accept without criticism the statement that "alcohol in some form . . . should be used in every case of typhoid fever from the beginning," for we prefer to wait until there is some indication for stimulation.

We regret to find that the authors are not able to give a better outlook in the treatment of tuberculosis. We cordially agree in their first point, that "the first indication for treatment is removal of the infected part, when possible." But we do not quite accept their next point, that "no known dose of any germicide which can be borne by the human system has any distinct power in even inhibiting the growth of the tubercle bacillus when once lodged in the body," and further, "it is possible that in the future a tubercular antitoxin may be produced, but, as tuberculosis is not a self-limited disease which produces an immunity, the prospects for success with its antitoxin do not seem brilliant." When the curability of phthisis has been so abundantly demonstrated, often by a spontaneously complete cure, the disease in other cases often becoming latent and quiescent, and from the *post-mortem* evidence in deaths from other causes as well as the clinical evidence during life, we may well question the dictum that the bacillus when once lodged in the body is positively indestructible, and we may further ask is it not likely that some form of drug treatment may accomplish results which are often obtained spontaneously? Creasote may not be an unfailing specific, it may not be a direct germicide, but no matter what its mode of action may be we have no doubt about its clinical value, and we think that it rightly holds its place and gains in favour.

As regards the specific action of the new tuberculin, it has been pointed out by Koch that immunity has commonly failed, because the bacilli are not absorbed and there is no real interaction between them and the tissues, but with the new fluid, in the making of which the dried cultures of the tubercle bacilli have been broken up mechanically by pounding in an agate mortar, it has been found that guinea pigs may be rendered immune in three weeks, virulent cultures no longer affecting them, and that a cure of tuberculosis in them may be obtained if the injections are begun within one or two weeks of infection. With these facts before us it would be unwise to speak or write despondently about the possible development of a valuable and useful antitoxin, even if it do not prove itself capable of accomplishing the impossible.

One other subject is worthy of passing notice. The infectious varieties of pharyngitis are mentioned, and the follicular form of tonsillitis is alluded to, but there is no general description of one of the most common diseases, and one which is exceedingly difficult to differentiate from diphtheria, the acute follicular streptococcus tonsillitis, which often appears to be contagious, and so often simulates diphtheria as to be incapable of differentiation without the bacteriological test. There are many interesting and perplexing points about this disease in which the young practitioner requires guidance, otherwise he adopts the simple and convenient method of calling them all diphtheria and of then publishing some astoundingly favourable results of the action of his favourite remedy.

On the whole this text book is a trustworthy guide, but we should have omitted the three pages of formulæ, most of which might be easily devised and improved.

